# A case report: using SNOMED CT for grouping Adverse Drug Reactions Terms

**DOI:** 10.1186/1472-6947-8-S1-S4

**Published:** 2008-10-27

**Authors:** Iulian Alecu, Cedric Bousquet, Marie-Christine Jaulent

**Affiliations:** 1 Université Paris Descartes, Faculté de Médecine; Inserm, U729; SPIM, Paris, 75006 France

## Abstract

**Background:**

WHO-ART and MedDRA are medical terminologies used for the coding of adverse drug reactions in pharmacovigilance databases. MedDRA proposes 13 Special Search Categories (SSC) grouping terms associated to specific medical conditions. For instance, the SSC "Haemorrhage" includes 346 MedDRA terms among which 55 are also WHO-ART terms. WHO-ART itself does not provide such groupings. Our main contention is the possibility of classifying WHO-ART terms in semantic categories by using knowledge extracted from SNOMED CT. A previous paper presents the way WHO-ART term definitions have been automatically generated in a description logics formalism by using their corresponding SNOMED CT synonyms. Based on synonymy and relative position of WHO-ART terms in SNOMED CT, specialization or generalization relationships could be inferred. This strategy is successful for grouping the WHO-ART terms present in most MedDRA SSCs. However the strategy failed when SSC were organized on other basis than taxonomy.

**Methods:**

We propose a new method that improves the previous WHO-ART structure by integrating the associative relationships included in SNOMED CT.

**Results:**

The new method improves the groupings. For example, none of the 55 WHO-ART terms in the Haemorrhage SSC were matched using the previous method. With the new method, we improve the groupings and obtain 87% coverage of the Haemorrhage SSC.

**Conclusion:**

SNOMED CT's terminological structure can be used to perform automated groupings in WHO-ART. This work proves that groupings already present in the MedDRA SSCs (e.g. the haemorrhage SSC) may be retrieved using classification in SNOMED CT.

## Background

WHO-ART (World Health Organization – Adverse Reaction Terminology) and MedDRA (Medical Dictionary for Drug Regulatory Activities) are the terminologies used in pharmacovigilance for case report coding and statistical data analysis. The generation of new knowledge on adverse drug reactions (also called signal detection) depends on the structure of the terminology. On the one hand, terminologies require terms as specific as possible in order to allow precise coding. On the other hand, signal detection commonly requires similar conditions to be recognized together in order to identify drug related problems [1-3]. In other words, precise coding requires a large number of (precise) terms, and statistical analysis requires a highly interconnected structure linking these terms. These requirements are highly interdependent. If the concepts are numerous but not sufficiently connected, statistical analysis will be done on sparse data and drug safety signals based on groupings will be more difficult to detect.

Medical problem oriented groupings have been manually introduced in MedDRA. Special search categories (SSC) are sets of MedDRA preferred terms related to a specific medical condition, for example "Upper gastro-intestinal bleeding conditions" or "Haemorrhage conditions". Recently, Standard MedDRA Queries (SMQ) replaced the SSCs. The SMQs have the same utility as the SSCs and are also manually defined.

WHO-ART does not provide such groupings of terms. The WHO-ART terminology has a hierarchical structure with restricted multiple inheritance, to avoid double counting of the same case through common drug surveillance (e.g. if the WHO-ART term corresponding to a drug safety report would be located in more than one unique category). The hierarchical structure is organized on three levels, but most of the terms appear on the first two levels as the second hierarchical level does not exist for two thirds of the coding terms. Sometimes terms with different levels of generalization may be siblings. As a consequence, grouping similar terms based on WHO-ART hierarchy will provide either very large clusters or very small ones not allowing tuning the focus of statistical analysis on specific or specifically related medical conditions.

In a previous paper we described a method for grouping WHO-ART terms related to common medical conditions [4]. The general idea was to propose a model to capture the meaning of WHO-ART terms within formal definitions in order to improve its hierarchical structure (and the grouping through querying). This model defined each term to be a synonym of a SNOMED CT concept. For example, the WHO-ART sibling terms "Thrombosis arterial" and "Thrombosis" have synonyms in SNOMED CT and these synonyms are related (i.e. "Thrombosis arterial" *is a *"Thrombosis"). Then, based on synonymy and relative position in SNOMED CT [5], two WHO-ART terms can be compared according to the generalization – specialization relation present in SNOMED CT (e.g. in WHO-ART the relation becomes "Thrombosis arterial" is a "Thrombosis").

In this previous experiment, we compared the MedDRA SSC (restricted to WHO-ART terms) to the groupings obtained automatically by classification based on the formal definitions of WHO-ART terms. WHO-ART terms present in SSCs were automatically retrieved. We were able to create grouping classes. For example, the medical search term "Disorders of Pregnancy" was automatically filled with several WHO-ART terms amongst which "Abortion", "Eclampsia", "Hydramnios", "Uterine atony" and "Uterine spasm". Most WHO-ART terms included in the MedDRA SSCs were automatically retrieved. However we found some drastic limitations due to the fact that these groupings are based only on a generalization – specialization relation and therefore limited to the high level grouping classes that the editors of SNOMED CT considered relevant (e.g. "Pain related disorders" but not "Pain located in digestive structure disorders"). For example, none of the 55 WHO-ART terms in the Haemorrhage SSC were found (e.g. "Haematoma", "Ecchymosis", etc.).

Consequently, the method needs to be improved to take into account groupings that rely also on associative relations such as "has location", "has morphology" or "has causative agent". A great effort has been done to make the meaning of medical concepts in SNOMED CT explicit for computers and this work is continued with every new release of SNOMED CT. For instance, all the pathologies associated to an inflammatory morphology are linked by the "has morphology" relation to the "Inflammation" concept. In the SNOMED CT hierarchy, the meaning of inflammation is furthermore divided in subclasses corresponding to different types of inflammation (e.g. necrotizing inflammation, acute inflammation, and chronic inflammation).

The objective of this work is to enrich the formal definitions of the WHO-ART terms by taking into account associative relations. We propose a Description Logic model corresponding to the following requirements:

1. The model should be populated with automatically extracted knowledge.

2. Classification of the model should retrieve clusters of medically related terms on given criteria.

We first describe the SNOMED-CT and WHO-ART terminologies. Then we present the logic description model for description of WHO-ART terms and the query mechanism. After presenting results of groupings using the new method we finally discuss the methodology to use for automated grouping and give an appraisal of the results.

## Materials

Our material includes the WHO-ART (2004 third quarter) and the SNOMED CT terminologies. SNOMED CT is available through UMLS (2005AA).

WHO-ART [6] is organized in three hierarchical levels. HLTs and PTs are linked to included terms (IT) that are synonymous or more specific than the former therefore we did not take into account the IT as hierarchical level.

1) The included terms (IT) and preferred terms (PT) level, which is recommended for the coding of adverse drug reactions (ADRs) and detailed drug safety statistical analysis.

2) High level term is a first grouping level. Only 31.3% of PTs are grouped in high level terms classes (HLT). The remaining PTs (68.7%) are linked directly to a system organ class (SOC). For example, the preferred term "Cardiomyopathy" is linked to the "Myo endo pericardial valve disorders" and "Body as a whole general disorders" SOCs.

3) At the most general level, PTs are grouped according to 32 system organ classes (SOC). SOCs group terms according to anatomy (e.g. "Gastrointestinal disorders") and/or public health medical problems (e.g. "Neoplasm").

The SOCs level is used for periodical statistical surveillance of national or international regulatory authorities. Under the SOC level, the polyhierarchy is forbidden in order to avoid double counting of an adverse reaction event. However a WHO-ART term can appear in up to three SOCs (one primary and two secondary).

As said earlier, clusters based on the native WHO-ART terminological structure (i.e. HLT, SOC) lead either towards very large semantic clusters (an average of 58 terms for SOCs) or very specific, granular ones (an average of 4 terms for HLTs).

SNOMED CT has a polyhierarchical structure and a large coverage of the medical domain [5] (approximately 350 thousand terms). It has a broader scope than coding ADRs. A large part of SNOMED CT consists of atomic concepts; they are finer-grained than the WHO-ART concepts and can be used as primitive concepts.

In a previous paper we generated an ontology based on the synonymy relationships between WHO-ART and SNOMED CT concepts [4]. The structure of SNOMED CT states the relative positions of the corresponding synonymous SNOMED CT concepts. This structure is based on the medical domain common sense and, in our case, it corresponds to the generalization – specialization relations represented as a hierarchy which, along with the synonymy relationship, will induce the generalization – specialization relations among WHO-ART concepts. In other words, we are relying on the SNOMED CT structure to discriminate WHO-ART terms.

At the implementation level, the classification task is based on the Description Logic (DL) principles. The Web Ontology Language (OWL DL) is a standard for knowledge representation that facilitates high machine interpretability. OWL provides existential quantification that allows one to describe, for example, the concept of "ADRs having a haemorrhage morphology" as "hasMorphology some Haemorrhage". Here, "hasMorphology" has the function of a role with "Haemorrage" as its role filler. The existential quantifier means that for each individual object that instantiates the expression "hasMorphology some Haemorrhage" there is at least one instance of the concept Haemorrhage related by the relation hasMorphology. For building the ontology, we have used Protégé as one of the most actively developed OWL enabled ontology editors [7], together with Racer, an inference engine performing automatic classification [8].

## Methods

In this section, we first present the main idea of our approach. It is centred around the synonymy relationship and the exploitation of the SNOMED CT hierarchy to support the reasoning process. Secondly, we present a methodology for acquiring formal definitions of WHO-ART terms and for querying the resulting model.

**Figure 1:**
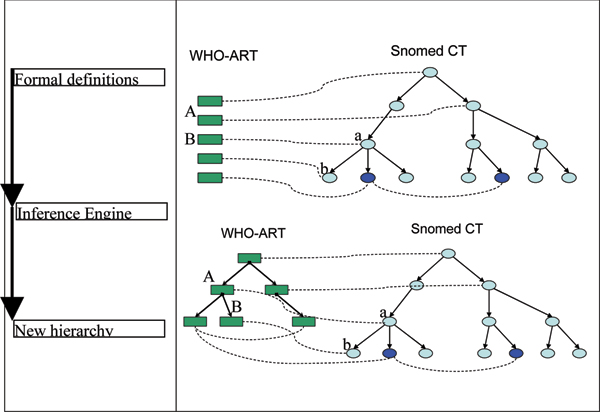
**Discrimination of WHO-ART terms by SNOMED CT hierarchy using the synonymy relationship**. The dark blue SNOMED CT concepts are both synonymous with the same WHO-ART term. A specialization link is introduced between the concepts A and B with regards to the a and b relative positions in the hierarchy.

The automated reasoning process within ontology is called subsumption and it relies on the set theory applied to possible instances of a certain concept. A concept *A *subsumes a concept *B *if and only if all the possible instances of *B *are also instances of *A*. The subsumption mechanism is preserved by the relationship "is synonymous". Therefore, if *A *"is synonymous" with *a*, *B *"is synonymous" with *b *and *a *subsumes *b *the inference engine will conclude that *A *subsumes *B *(see Figure 1).

The "synonymy" relationship as defined above is a structuring relationship when the target terms of the relation (fillers; i.e. *a *and *b *in the previous example) belong already to a hierarchy. The hierarchical relations in SNOMED CT induce a *discriminative *property to the synonymy relation. Based on that, the defined WHO-ART concepts can be placed in a hierarchy (see Figure 1).

Both SNOMED CT and WHO-ART are included in the Metathesaurus of the Unified Medical Language System (UMLS). Here, a concept is build based on the synonymy relation between terms coming from different source vocabularies. In our ontology, the synonymy relations are those extracted from UMLS [3].

Sometimes terms not considered synonymous by the SNOMED CT editors can be part of the same UMLS concept (e.g. "Aplastic anemia", "Aplastic anemia NOS" and "Hematopoietic aplasia"). This is due to the fact that SNOMED CT is more granular than most of the UMLS sources.

Within the UMLS Metathesaurus, a WHO-ART term has a small number of SNOMED CT synonyms. An ideal synonymy relation would be a "one-to-one" relationship between a WHO-ART concept and a SNOMED CT concept. The "one-to-one" or "one-to-few" relationship is very important in our model where the discriminative power is given by the SNOMED CT hierarchy (Figure 1).

Ensuring consistent synonymy across millions of concepts in the UMLS Metathesaurus is difficult and depending on use sometimes not appropriate [9]. Several SNOMED CT concepts may be proposed as synonyms of the same WHO-ART concept although they have different meanings. This fact raises some modelling issues on the logical operators (AND/OR) which stand between the different asserted synonymous terms. In subsumption, which is defined as a strict set inclusion condition, these logical operators act like the intersection/union (AND/OR) operators. Assuming a perfect synonymy, the operator would not matter: if *a *synonym to *b *then *a *≡ *b *as well as (*a *⋂ *b*) ≡ (*a *⋃ *b*).

In the present work, we use "OR" between SNOMED CT synonyms of the same WHO-ART term in order to capture all the meanings of a WHO-ART concept in the formal definition.

Besides differentiating WHO-ART concepts, SNOMED CT provides multiple inheritance of concepts. SNOMED CT makes possible the grouping of WHO-ART concepts thanks to the high level categories (*grouping *classes), however limited to the ones that the editors of SNOMED CT considered relevant (e.g. in SNOMED CT there is a "Pain related disorders" grouping, but not "Hemorrhage related disorders" [3]).

The methodology is organized in three steps. During the first step, the synonymous SNOMED CT terms are extracted from the UMLS Metathesaurus and linked to corresponding WHO-ART concepts. The first step is fully described in our previous work [4] and has been shortly presented in the current section. The second and third steps are described in the following sections. During the second step, associative relations are added in order to refine the synonym-based ontology. The last step involves the methodology for querying the model and grouping terms according to given criteria.

### Refinement of synonymy-based ontology

The synonym-based ontology is used as a starting point for the addition of the associative relationships (e.g. relations like "has morphology" are *associative *relations). When a large amount of concepts are related to one or few concepts, the associative relations have a grouping role (e.g. there are numerous inflammatory medical conditions and they are all related to "Inflammation" itself or to specializations of "Inflammation": acute inflammation, etc.). Combining the *associative relations *and the *primitive grouping concepts *(e.g. inflammation) is a very flexible way to get different situation-tailored clusters of terms that can be very useful for data retrieval.

The same relation having multiple objects (e.g. Mallory-Weiss syndrome has "Laceration" and "Haemorrhage" as object for "has associated morphology") raises the question of what logical operator to choose to relate them. We chose the "AND" operator in order to create a multiple inheritance (e.g. Mallory-Weiss syndrome will be both in "Laceration related disorders" as well as in "Haemorrhage related disorders"). This choice is motivated by the fact that "AND" is computed as an intersection operation on sets and that the subsumption is inferred based on the strict inclusion operator on sets. An intersection of sets will always be composed by fewer or the same number of elements than the union (e.g. "OR"). In our ontology a more restricted (e.g. "AND") concept is more probable to be STRICTLY included in a grouping concept. This allows the grouping of WHO-ART concepts using the SNOMED CT concepts as grouping criteria.

In our model, we have used only the relations "has finding site" and "has associated morphology" which are both extracted from SNOMED CT. The other SNOMED CT associative relations (e.g. "has_onset", etc.) are not of any interest at this time in our model. For each WHO-ART concept a formal definition is automatically generated as follows:

*C*^*who*^* complete*

*(is_syn some (C*^*snmct*^*or C*^*snmct*^*or ....))*

*or (has_finding_site some (C*^*snmct*^*and C*^*snmct*^*and ....))*

*or (has_associated_morphology some (C*^*snmct*^*and C*^*snmct*^*and ....))*

In the previous definition, *C*^*who*^ is a WHO-ART concept and *C*^*snmct *^is a SNOMED CT concept.

For example, the formal definition [10] of "Gastritis" and "Gastritis acute" is:

*Gastritis** complete*

(is_syn some (SNMCT:Gastritis))

or (has_finding_site some (SNMCT: Stomach_structure))

or (has_associated_morphology some (SNMCT:Inflammation))

*Gastritis acute** complete*

(is_syn some (SNMCT:Acute_Gastritis))

or (has_finding_site some (SNMCT:Stomach_structure))

or (has_associated_morphology

some (SNMCT:Acute_Inflammation))

Given the fact that the "Gastritis acute" is subsumed by "Gastritis" and "Inflammation acute" is subsumed by "inflammation" in SNOMED CT, the reasoning process will classify WHO-ART corresponding terms "Gastritis acute" as a subclass of "Gastritis".

### Querying the model

Making queries on the presented model raises the problem of managing missing data, precisely missing role fillers.

In this work, we are filling our model with automatically extracted information and we assume that the formal definitions are not complete. By choosing this way to write the formal definitions we are taking full profit from the open world assumption (OWA). Roughly speaking, we are telling to the inference engine that the defined concept has the properties we are asserting but also that it can have any other property. In OWA, this notion is expressed by "nothing" meaning that the logical value (i.e. "true" or "false") is unknown and being equivalent with the empty set in the set theory. The empty set will be subsumed by any class. In a closed world system anything unknown to the system gets the value "false".

For example, the "obesity" term has no fillers for the relations "has morphology" and "has localisation". The reasoner will assume "nothing" as filler.

In our context, a restriction to "anything" (*owl:thing*) is given by default each time a WHO-ART concept does not have a filler for a relationship. In other words, any restriction on the relationship is removed and we are asserting that the object is somewhere in the model.

**Figure 2:**
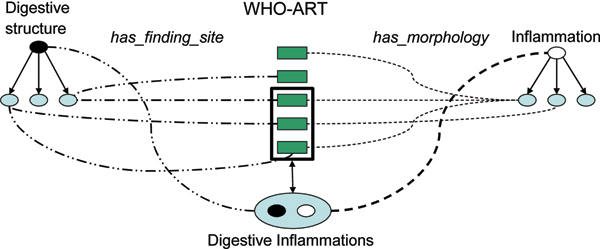
An example of querying the model.

Following this principle, the query class that subsumes (and therefore groups) all digestive inflammations will be defined as (Figure 2):

Digestive inflammations

(is_syn some (owl:thing))

or (has_finding_site some (SNMCT:Digestive_structure))

or (has_associated_morphology some (SNMCT:Inflammation))

In the above definition, the query "Digestive inflammations" is synonymous with *owl:thing *It does not mean that the "Digestive inflammations" class subsumes (in terms of mathematical set theory: *includes*) all the classes in the ontology. In fact, it will subsume the concepts synonymous to *owl:thing *located in some kind of digestive structure and which has some kind of inflammation as their associated morphology.

To illustrate this point, we can easily find a class defined as synonymous to something, which would have an associated inflammatory morphology but which would not be located in a digestive structure (e.g. "Vasculitis"). It is obvious that this class will not be included in "Digestive inflammations", but in another defined clustering concept. If we do not systematically define the missing fillers as *owl:thing*, this will lead to false situations.

For instance, without a value for "has finding site" and "has associated morphology", "Obesity" would be subsumed by "Digestive inflammations".

Obesity complete

(is_syn some (SNMCT:Obesity or SNMCT:Obese))

The figure 2 illustrates behaviour of a query in our model.

## Results

Using the technique presented in the previous sections, 85.9% (1,597) of WHO-ART terms were successfully mapped through UMLS to one or more SNOMED CT synonyms.

The ontology contains 8,454 classes including 1,597 defined classes involving 4,482 existential assertions. The primitive concepts hierarchy represents 1.9% of SNOMED CT (366,170 concepts). It takes an average time of 4 minutes for RacerPro [11] to classify this ontology.

The resulting WHO-ART structure is evaluated against the SSCs built by MedDRA (7.1) editors [12] (available through UMLS). For evaluation purposes, the MedDRA SSCs are restricted to the terms that are also in the WHO-ART terminology. These restricted SSCs will be further referred to as MedDRA SSC.

In order to illustrate the results of our method, we focus here on the results concerning the "Hemorrhage" SSC as an example of grouping class based on associative relations. The automatic grouping relying only on the synonymy based ontology is missing in many WHO-ART terms. Indeed, for this SSC there is no higher class grouping in SNOMED CT, therefore we define a query as:

*Hemorrhage_related*

(is_syn some (owl:thing))

or (has_finding_site some (owl:thing))

or (has_associated_morphology some (SNMCT:Hemorrhage)).

We obtain a class containing 46 of the 55 WHO-ART concepts mapped to corresponding MedDRA Hemorrhage SSC concepts, 3 are not mapped (14% of WHO-ART terms are not mapped to SNOMED CT) and the remaining 6 are concepts for which the relation to "Hemorrhage" is not in SNOMED CT.

There are 66 WHO-ART terms regrouped in this class. The false positives include terms like "Duodenal ulcer hemorrhagic and perforated", "Colitis hemorrhagic", "Purpura" or "Hemorrhage in pregnancy" that are in different forms in the original MedDRA SSC's and that were not mapped correctly as they have different lexical forms in the original MedDRA SSCs.

The "Hemorrhage" example shows a preliminary validation of the method. In the same way, we plan to create other query classes as long as they can be characterized using the primitive grouping concepts and associative relations.

## Discussion

Cimino argued that terminologies should comply with desiderata such as formal definitions and polyhierarchy [13]. This paper presents a method to transform the WHO-ART terminology from a classification system with no formal inference to a knowledge based nomenclature enabled with inference capabilities. Moreover, it presents an incremental method able to improve and make evolve the model.

This is achieved by reusing an existent resource [14] rather than building the ontology from scratch. The method benefits from the fact that SNOMED CT embeds in its hierarchy different semantic views of each term and provides detailed definitions for each of its terms. Our main achievement is that we obtain meaningful groups of terms using automated classification; the relevance of these groups is compared to the manually built SSCs.

Based on the hierarchy of SNOMED CT only, we had obtained relevant grouping compared with SSCs [3]. Yet the automatically generated SSCs based on associative relations had been incomplete. Our hypothesis for the present work has been that this limitation can be improved by adding previously ignored knowledge from SNOMED CT. The main issue was the addition of this knowledge in the model.

The 85.9% success of the mapping step is still insufficient but could be naturally improved since the UMLS Metathesaurus is reviewed and updated regularly. To improve this percentage independently of the review frequency of the UMLS, we consider investigating approximate matching by browsing other terminologies in UMLS. In further work NLP techniques will be considered also.

The mappings provided by UMLS present some limitations; terms can be considered synonymous although they reside on different axes in SNOMED CT (e.g. "Thrombosis", a "Qualifier value" and "Thrombotic disorders", a "Clinical finding").

The synonym-based method was successful for the SSCs that have corresponding classes in SNOMED CT (e.g. "Pain" mapped to "Disorder characterized by pain"). The presented method improved the results for the other SSCs such as "Haemorrhage", where the groupings rely more on associative relations than on "*is_a*" relations.

The resulting terminological resource can be considered an evolving one as it allows queries that could give feedback on synonymous and associative relationships. For example, for "Menorrhagia" that has not been found in the "Hemorrhage related" class one can easily add "Hemorrhage" as object for the "has associated morphology" relation.

## Conclusion

UMLS already interconnect medical terminologies and allows performing transcoding and mappings between almost all of the medical terminologies actually in use. The internal organisation, more precisely the rapports that the authors establishes between the concepts in all these terminologies meets the most frequent statistical data analyses needs. As some of these data aggregation requirements are common between different medical fields, the queries (i.e. term groupings) can be performed not only by using the coding terminology but also the terminologies related to the one used to code data. This strategy is greatly helped by UMLS that allows in a very simple way to transcode data.

In this work, we showed that SNOMED CT terminological structure can be used to perform automated groupings in WHO-ART. This work proves that groupings already present in the MedDRA SSCs (e.g. the haemorrhage SSC) may be retrieved using classification in SNOMED CT.

Further work will test the assumption that SNOMED CT can increase the chances to find earlier drug safety signals from case reports differently coded but reporting on a same medical issue. Moreover, this technique can be very useful to refine a terminology structure, to audit it and propose improvements like for example add terms for concepts that are not represented or detect incoherence.

## Authors' contributions

IA: built the resource, performed the classification algorithm and wrote the first draft of this paper.

CB: performed an initial appraisal of the WHO-ART and MedDRA terminologies that showed the interest of building formal definitions for pharmacovigilance.

CB, MCJ: The three authors participated in the initial design of the study, in the review of the results and the editing of the article.
